# The largest human cognitive performance dataset reveals insights into the effects of lifestyle factors and aging

**DOI:** 10.3389/fnhum.2013.00292

**Published:** 2013-06-20

**Authors:** Daniel A. Sternberg, Kacey Ballard, Joseph L. Hardy, Benjamin Katz, P. Murali Doraiswamy, Michael Scanlon

**Affiliations:** ^1^Lumos Labs Inc.San Francisco, CA, USA; ^2^Combined Program in Education and Psychology, University of MichiganAnn Arbor, MI, USA; ^3^Department of Psychiatry and Duke Institute for Brain Sciences, Duke UniversityDurham, NC, USA

**Keywords:** lifestyle factors, learning, aging, cognition, cognitive enhancement, fluid intelligence

## Abstract

Making new breakthroughs in understanding the processes underlying human cognition may depend on the availability of very large datasets that have not historically existed in psychology and neuroscience. Lumosity is a web-based cognitive training platform that has grown to include over 600 million cognitive training task results from over 35 million individuals, comprising the largest existing dataset of human cognitive performance. As part of the Human Cognition Project, Lumosity's collaborative research program to understand the human mind, Lumos Labs researchers and external research collaborators have begun to explore this dataset in order uncover novel insights about the correlates of cognitive performance. This paper presents two preliminary demonstrations of some of the kinds of questions that can be examined with the dataset. The first example focuses on replicating known findings relating lifestyle factors to baseline cognitive performance in a demographically diverse, healthy population at a much larger scale than has previously been available. The second example examines a question that would likely be very difficult to study in laboratory-based and existing online experimental research approaches at a large scale: specifically, how learning ability for different types of cognitive tasks changes with age. We hope that these examples will provoke the imagination of researchers who are interested in collaborating to answer fundamental questions about human cognitive performance.

## Introduction

While many scientific fields ranging from biology to the social sciences are being revolutionized by the availability of large datasets and exponentially increasing computational power, the dominant approach to studying human cognitive performance still involves running small numbers of participants through brief experiments in the laboratory. This approach limits the kinds of questions that can be practically studied in important ways. For one, most studies depend on a convenience sample of university undergraduates, limiting the broad applicability of findings (Heinrich et al., 2010). The need for research participants to return to the laboratory also limits the ability to study fundamental questions about the variables that influence learning over time and across the lifespan.

Understanding how demographic and lifestyle factors influence cognitive function has important health and policy implications. These questions are often difficult to examine using laboratory-based approaches because they require the experimenter to recruit sufficient numbers of participants across a wide range of demographic backgrounds. Studies of how cognitive performance changes with age tend to compare a sample of university undergraduates to older adults, and as a result can only tell us about the discrete differences between these samples. Since age varies continuously in the population, determining the rate at which performance and learning change with age across the lifespan would require studying a large number of participants across a continuous range of ages. This type of study would be prohibitively time-consuming and expensive to run in a conventional psychology laboratory. Likewise, even the largest observational or multi-center controlled clinical trials examining effects of various interventions on cognitive performance have generally consisted of no more than several thousand individuals from restricted geographic and demographic backgrounds—e.g., Whitehall II *N* = 10,314 (Marmot et al., 1991) Women's Health Initiative Memory Study *N* = 8,300 (Craig et al., 2005).

## The lumosity platform and dataset

Given the limitations of conventional approaches, it is worthwhile to consider alternative methods to gathering data on human cognitive performance. With the rise of the Internet, web-based research in the behavioral sciences has become more common, particularly in studies of human cognition (Reips, 2004). While concerns remain, the potential of web-based research to recruit larger samples from a wider variety of demographic backgrounds has been widely acknowledged (Kraut et al., 2003; Birnbaum, 2004; Skitka and Sargis, 2006).

**Figure 1:**
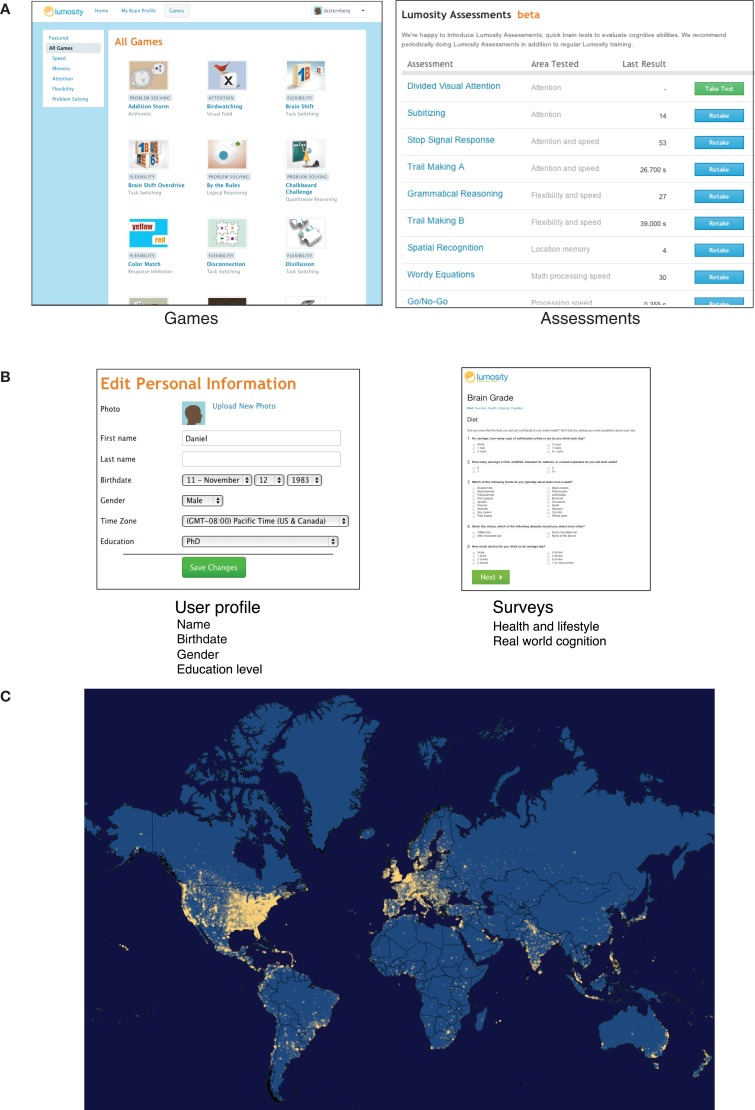
**(A)** Lumosity includes exercises designed to improve cognitive performance targeting five areas of cognition, along with assessments based on standard neuropsychological tasks. **(B)** Demographic information is available from users' profiles and surveys that users can choose to participate in. **(C)** A map of users' locations based on their IP address at last login. The map was generated from a database of user IP addresses at login. Approximate Latitude and longitude coordinates were obtained for each IP address using MaxMind's GeoLiteCity database (available at http://www.maxmind.com/app/geolitecity). These coordinates were then rounded to the nearest 1/100th of a degree and aggregated to obtain a count of the number of users at each rounded coordinate. The size of each dot was mapped to the floor of the base-10 log of the number of users. As IP addresses were missing for some users, and in some cases IP addresses could not be mapped to geographic coordinates, the data used to generate the map was based on the geographic coordinates for 15,162,193 users.

Lumosity is a web-based cognitive training platform that includes a suite of cognitive training exercises, assessments, and an integrated training system designed for the purpose of improving users' cognitive abilities. As the user base has grown rapidly over the past six years, the database of users' cognitive performance has become the largest dataset of human cognitive performance to our knowledge. As of January 23, 2013, the dataset includes 36,140,947 users representing 231 distinct ISO-3166 country codes. These users have trained on the cognitive exercises 609,017,147 times and taken online neuropsychological assessments 6,661,302 times (see Figure 1A for screenshots of the game and assessment pages).

In addition to engaging in training tasks and taking assessments, users voluntarily provide demographic information, including their age, gender, and level of education. They also have the opportunity to participate in a number of surveys about health, lifestyle, and real-world cognitive activities (Figure 1B). A user's location can be roughly determined from his or her IP address, which allows researchers to relate approximate geographic information to cognitive performance and to measure geographic reach (Figure 1C).

While internal research using this growing dataset has been ongoing for some time, Lumos Labs has recently begun to work with outside researchers who are also interested in analyzing cognitive performance at large scale, as one arm of the *Human Cognition Project* (HCP), a collaborative research program to understand the human mind. External researchers interested in analyzing de-identified portions of the dataset apply through the HCP website (http://hcp.lumosity.com). As part of the application process, researchers are asked to present a specific analysis plan. The Lumos Labs research and development team, and in some cases, external research advisors, vet proposals based on the quality of the specific analysis plan. All well-designed proposals are accepted. Lumos Labs allows researchers to publish any findings following from the accepted analysis plan without requiring further consultation with the company. At this time, the large majority of ongoing projects analyzing the Lumosity dataset are focused on basic psychological phenomena that are not directly related to validating cognitive training.

Here, we present two initial demonstrations of the power afforded by examining human cognitive performance at large scale. In the first example, we examine how cognitive performance relates to general health and lifestyle factors, based on a large survey of hundreds of thousands of users from the dataset. In the second example, we look at how task improvements change with age, and how these age-related changes differ for tasks that depend on different cognitive abilities.

## Example 1: Health, lifestyle, and cognitive performance

Many lifestyle factors have been shown to influence cognitive abilities, and a cognitively active lifestyle has been linked to reduced levels of potential precursors to dementia (Landau et al., 2012) and a reduced likelihood of developing dementia (Doraiswamy, 2012). For these reasons, we were interested in whether users' initial performance correlated with their self-reported lifestyle habits. In order to examine this question, we designed a survey of health and lifestyle habits that has now been taken by millions of individuals across the world (available at: http://www.lumosity.com/surveys/brain_grade). Here, we focus on two particularly interesting questions about lifestyle habits from this survey that vary continuously in the population: sleep and alcohol consumption. These variables have been included in other surveys that also measured cognitive function (e.g., Marmot et al., 1991), and we were interested in whether the influence of these variables on performance in our user base would correspond to what has been observed in the existing literature.

### Methods and materials

We obtained survey data for all users who took the health and lifestyle survey between March 2011 and January 2012. For each of these users, we also obtained their initial scores on three cognitive exercises, where available. These exercises were chosen for reliability as well as coverage: they are some of the most popular training tasks, are shown within the first few days of training, and represent distinct cognitive abilities. The three exercises are described below.

*Speed Match* is a one-back matching task in which users respond whether the current object matches the one previously shown. Users respond to as many trials as they can in 45 s. We used the number of correct responses the user made before the end of the task as the measure of performance.

*Memory Matrix* is a spatial working memory task in which users are shown a pattern of squares on a grid, and must recall which squares were present following a delay. The tasks uses a variant of a one-up one-down staircase method (Levitt, 1971) in order to find the user's memory threshold. We used this threshold as the measure of performance.

*Raindrops* is a speeded arithmetic calculation task in which new arithmetic problems continuously appear at the top the screen inside of raindrops. Users need to answer the problems before the raindrops reach the bottom of the screen. Once three raindrops have reached the bottom of the screen, the task ends. We used the number of correct responses made before the task ended as the measure of performance.

### Results

**Figure 2:**
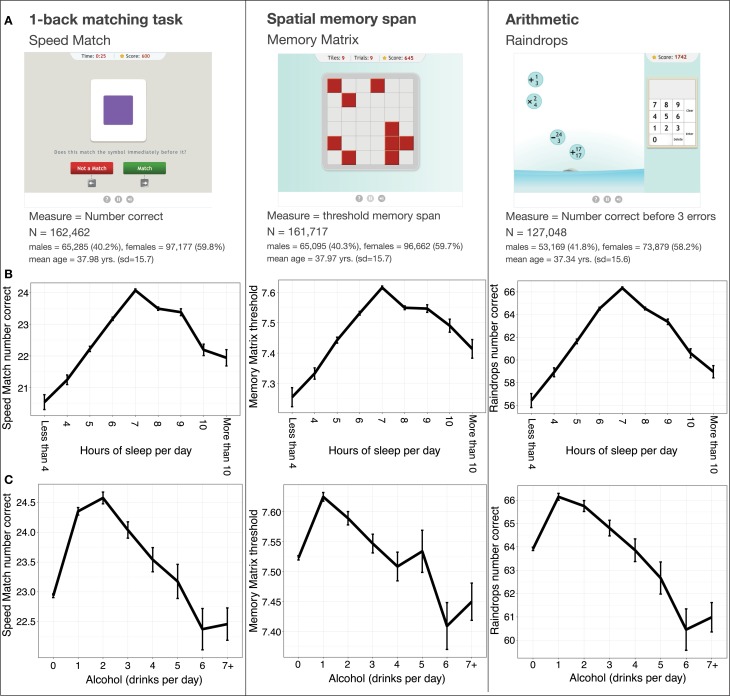
**(A)** Exercises used in the analysis of the health and lifestyle survey. **(B)** The effect of reported sleep on game performance. **(C)** The effect of reported alcohol intake on game performance (controlling for age, gender, and level of education).

Figure 2A provides sample sizes and demographic information from the three tasks. For each task, the relevant measure was first fit to a general linear model including age (up to 4th degree polynomial), level of education (approximate years), gender, and the interactions of these variables as predictors. In the case of Speed Match and Raindrops, where the relevant measure was the number of correct responses, the model included a Poisson link function in order to capture the distribution. The residuals returned by each model were used as the dependent measure for the further analyses.

The main effects of self-reported sleep and alcohol intake were measured for each task via separate multivariate linear regression models. These models revealed positive linear effects of hours of sleep for and negative quadratic effects of sleep for all three tasks (see Table 1 for model coefficients and relevant statistics). More specifically, we found that cognitive performance in all three tasks was greater for users reporting larger amounts of sleep up to 7 h per night, after which it began to decrease (Figure 2B). The models also revealed significant negative linear and negative quadratic effects of alcohol for all three tasks. Low to moderate alcohol intake was associated with better performance in all three tasks, with performance peaking at a self-reported 1 or 2 drinks per day, depending on the task (Figure 2C), and decreasing as alcohol intake increased from there. The presence of negative quadratic effects for both predictors indicated that the effects of sleep and alcohol intake on performance had an inverted U-shape.

**Table 1:** **Model coefficients and t-statistics for the linear and quadratic effects of reported hours of sleep and alcohol intake, taken from the grand regression model**.

**Variable**	**Speed match**	**Raindrops**	**Memory matrix**
Sleep (linear)	B = 1.30	B = 1.47	B = 0.17
	*t* = 6.33^***^	*t* = 3.29^*^	*t* = 6.90^***^
Sleep (quadratic)	B = −2.83	B = −8.25	B = −0.28
	*t* = −14.77^***^	*t* = −19.74^***^	*t* = −12.3^***^
Alcohol (linear)	B = −1.40	B = −4.96	B = −0.14
	*t* = −6.36^***^	*t* = −10.5^***^	*t* = −5.46^***^
Alcohol (quadratic)	B = −1.37	B = −2.19	B = −0.07
	*t* = −6.96^***^	*t* = −5.22^***^	*t* = −3.01^*^

* p < 0.01,

^**^p < 0.001,

*** *p < 0.0001*.

### Discussion

The associations between sleep, alcohol intake, and cognitive function observed here are comparable to previous findings from the Whitehall II study. An analysis of Whitehall II participants also found that those who reported around 7 h of sleep showed the highest cognitive performance on a battery of psychological assessments (Ferrie et al., 2011). Another study of the same cohort found that alcohol intake reduces the likelihood of poor cognitive function (Britton et al., 2004), though this study did not observe the same reduction in cognitive performance at higher levels of consumption that we found in our analysis. One possible explanation for this difference is that Britton and colleagues focused on whether a participant's cognitive performance scored in the bottom quintile, as a measure of “poor cognitive function,” while our analysis looked at the average performance at each level of alcohol consumption. The increased scale of our dataset may have allowed us to observe this non-linearity in the dose-dependent effects of alcohol consumption. Other unobserved demographic covariates may also provide some explanation for the divergent findings, as the Whitehall II cohort is also restricted to civil service workers from the United Kingdom, while Lumosity users come from a wide range of demographic backgrounds and are located all over the world.

As these findings are correlational in nature, there may be other related but unobserved variables that explain some of the effects of alcohol consumption and sleep in our data. For example, the apparent cognitive advantage for those who report moderate alcohol intake may be in part due to increased social and cognitive engagement compared to those who report little or no alcohol consumption. Thus, while we would not want to strongly assert that the real causal effects of these variables exactly mirror our findings, these results instead provide a rough profile of the habits of individuals who tend to show higher cognitive function that can be filled in as we obtain additional health and lifestyle data. This first example should also serve as a testament to the ability to quickly obtain reliable data from a large numbers of individuals using the survey platform, as we were able to gather all of this data solely from new users who had joined the site within a 9-month period.

## Example 2: Cognitive task improvements and aging

While aging researchers have discovered a great deal about how baseline performance declines with age for different cognitive abilities (Park, 1999; Salthouse, 2009) less is known about how the ability to learn different kinds of skills changes over the lifespan. Exploring this question using standard laboratory-based approaches would require recruiting a large number of participants across a wide range of ages and bringing them into the lab to perform multiple tasks many times over the course of weeks or months. Existing web-based approaches also face their own difficulties in studying learning over time. Other platforms that have recently become popular for running psychology studies on the web, such as Amazon Mechanical Turk (Buhrmester et al., 2011; Mason and Suri, 2012), are poorly suited for running the multi-session studies necessary to obtain this type of data, and even very recent work measuring cognitive performance across the lifespan at a relatively large scale has to date only examined baseline performance (Hampshire et al., 2012).

This type of data may be difficult to obtain via other web-based platforms in part because, while it is relatively simple to use small payments to individuals and/or online advertising to quickly obtain baseline cognitive performance data from a large number of individuals, there is little incentive for participants to return on a regular basis. In contrast, Lumosity users are specifically interested in cognitive training and are able to train on a large variety of cognitive tasks as often as they would like. As a result, they commonly return regularly over the course of months and years. These unique characteristics make it possible to examine how learning ability changes year by year over the lifespan, and how aging might affect learning differently across distinct cognitive abilities. As a preliminary demonstration of the ability to measure these differences in this dataset, we looked at how a user's age influences how much he or she improves over the course of the first 25 sessions of a cognitive task, and compared tasks that rely on abilities linked to fluid intelligence, such as working memory tasks, vs. those that rely more on crystallized knowledge, such as verbal fluency and basic arithmetic.

### Methods and materials

In order to test for differential effects of aging on improvement in fluid intelligence and crystallized knowledge tasks, we chose four particular exercises in our database, two of which rely on working memory, a known correlate of fluid intelligence, and two that rely on declarative knowledge—verbal fluency and basic arithmetic. For each of the four tasks, we pulled the first 25 sessions for all user who had trained on that task at least 25 times. (minimum *N* = 22,718). Two of the tasks (*Memory Matrix* and *Raindrops*) were the same as those used in the health and lifestyle analysis. For these tasks, the same measures that were used in the health and lifestyle analysis were used. The other two tasks are described below.

*Memory Match* is a 2-back working memory task in which users respond whether the current object matches the one shown two trials ago. Users respond to as many trials as they can in 45 s. The relevant measure of performance in this task was the number of correct responses users made.

*Word Bubbles* is a verbal fluency task in which users type as many words as possible matching a particular word stem, with the constraint that their score depends on making multiple words of different lengths. The task lasts 3 min. The relevant measure of performance was the number of correct responses users made.

**Figure 3:**
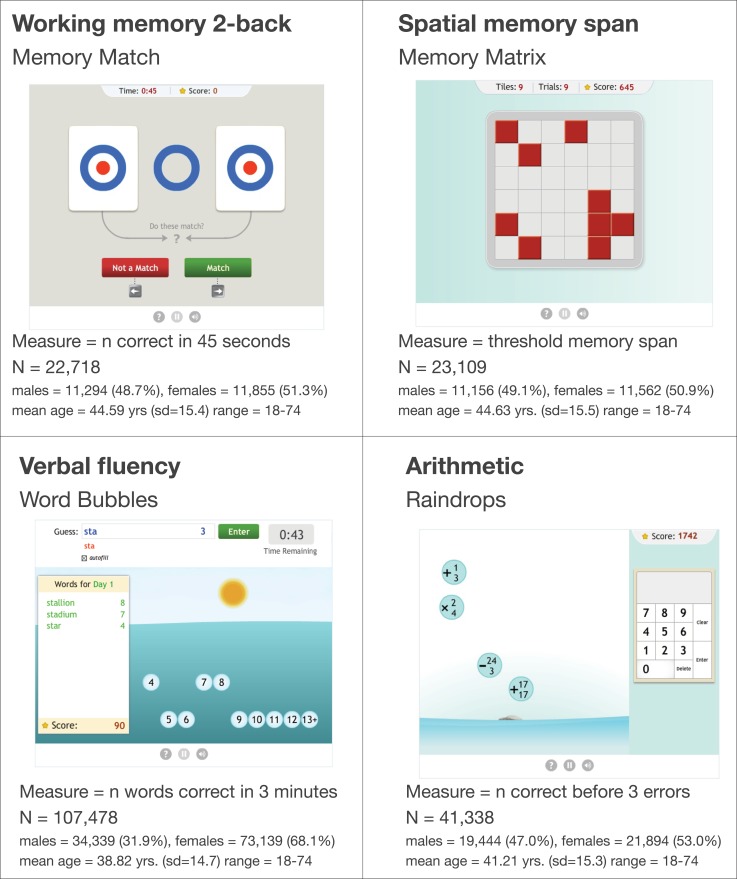
**The four exercises used in the aging and learning analysis, and demographic information for each game**.

Figure 3 shows sample sizes and demographic information for each task. In order to compare performance across the tasks on the same scale, the dependent variable for each task was transformed via a scaling table obtained by applying an inverse rank normalization procedure to a separate dataset of baseline raw scores from a large number of users (range = 93,832-3,600,595). For each task, the empirical percentile for each baseline raw score was mapped to its corresponding place on a normal distribution with μ = 100, σ = 15.

For at least one task (Word Bubbles), the mean baseline score for users who trained at least 25 times was greater than 100, indicating that the subset of users who chose to train 25 times had higher baseline scores than the average user who may have trained only once. In the analysis below, the main effects of the variables contrasting individual tasks at baseline should control for any differences between the average baseline score for each task.

### Results

We examined the effect of age on performance at baseline and on learning across tasks using linear mixed effects model predicting the scaled score based on the user's age level of education, gender, task, and session (1st vs. 25th). The model also included interaction terms for gender × task, education level (approximate years) × task, session × task, age × task, and age × task × session. A quadratic effect of age was also included in the model, along with the same respective interactions with task and time. Continuous covariates (age and education) were centered before inclusion in the model. The model also included a separate random intercept for each user.

The task variables in the model were coded using the following planned orthogonal contrasts. Contrast 1 compared the “fluid intelligence” tasks (Memory Matrix, Memory Match) to the “crystallized knowledge” tasks (Raindrops, Word Bubbles). Contrast 2 compared Memory Match and Memory Matrix. Contrast 3 compared Raindrops and Word Bubbles. Gender, education, and their interactions with the task contrasts were included in the model specifically to control for known effects of these variables. Table 2 gives the coefficients and t-statistics for the fixed effects in the model.

**Table 2:** **Coefficients and *t*-statistics for age and learning mixed effects model**.

**Predictor**	**β**	***t***
Age (linear)	**−0.318**	−94.6^***^
Age (quadratic)	**−0.003**	−11.7^***^
Education (1–7, some high school – PhD)	**0.957**	32.7^***^
Gender (Male = 1, Female = −1)	**0.558**	12.8^***^
C1: Fluid (1,1) vs. Crystallized (−1,−1)	**−3.887**	−83.5^***^
C2: Memory Match (1) vs. Memory Matrix (**−**1)	**−0.934**	−12.3^***^
C3: Raindrops (1) vs. Word Bubbles (**−**1)	**−1.807**	−35.6^***^
Session [*t*_(1)_ = 0, *t*_(25)_ = 1]	**15.8**	265.1^***^
C1 × Age (linear)	**−0.079**	−32.9^***^
C2 × Age (linear)	**0.017**	4.3^***^
C3 × Age (linear)	**−0.063**	−26^***^
C1 × Age (quadratic)	**0.003**	20.3^***^
C2 × Age (quadratic)	**0.001**	4.7^***^
C3 × Age (quadratic)	**−0.0005**	−2.7^**^
C1 × Education	**−0.494**	−28.9^***^
C2 × Education	**−0.171**	−6.3^***^
C3 × Education	**0.235**	12.5^***^
C1 × Gender	**0.297**	11.4^***^
C2 × Gender	**−0.172**	−4.1^***^
C3 × Gender	**1.577**	56.4^***^
C1 × Session	**1.546**	25.9^***^
C2 × Session	**5.468**	54.2^***^
C3 × Session	−0.106	**−**1.7
Session × Age (linear)	**−0.099**	−34.2^***^
Session × Age (quadratic)	**0.001**	**4.8**
C1 × Age (linear) × Session	**−0.057**	−19.5^***^
C2 × Age (linear) × Session	**−**0.006	**−**1.3
C3 × Age (linear) × Session	0.004	1.2
C1 × Age (quadratic) × Session	0.000	**−**1
C2 × Age (quadratic) × Session	**−0.003**	−8.0^***^
C3 × Age (quadratic) × Session	**−0.001**	−5.4^***^

*The model was fit using the lmer function, part of the lme4 package in R. Significance values are based on highest posterior density intervals derived from 10000 Markov Chain Monte Carlo samples, using the pvals.fnc function in R's languageR package*.

^*^p < 0.01,

** p < 0.001,

*** *p < 0.0001*.

We observed negative age-related differences in performance on all tasks, as indicated by the negative linear coefficient for age. However, the presence of an interaction of age and the crystallized/fluid game contrast indicates that performance on the tasks that rely on fluid intelligence decreased with increasing age at a faster rate than the tasks that rely on crystallized intelligence. The negative coefficient for the fluid/crystallized tasks contrast with the quadratic effect of age suggests that the negative age-related effect of age on the fluid intelligence tasks started earlier and leveled off compared to the crystallized intelligence tasks, which were preserved for longer before beginning to decrease. Looking within the two tasks types, the negative linear age-related difference was steeper for Memory Match than for Memory Matrix, and was steeper for Raindrops than for Word Bubbles. These additional interactions may provide some indication of the relative importance of fluid and crystallized knowledge and/or processing speed in these tasks.

In general, users improved with training, as indicated by the main effect of session. The significant interaction of session with the first two game contrasts reveals that users improved more at the fluid intelligence tasks than the crystallized knowledge tasks, and improved most at Memory Match. The amount of improvement between sessions decreased as age increased, and this negative effect of aging on learning was greater for the tasks that relied on fluid intelligence than those that relied on crystallized knowledge, as indicated by the strong three-way interaction of this contrast with the linear effect of age and training session. Finally, the relatively large three-way (game × quadratic age × session) interaction for the second task contrast suggests that the reduction in improvement in older adults began later and accelerated for Memory Match compared to Memory Matrix, where the reduction in improvement began earlier before leveling off.

### Discussion

**Figure 4:**
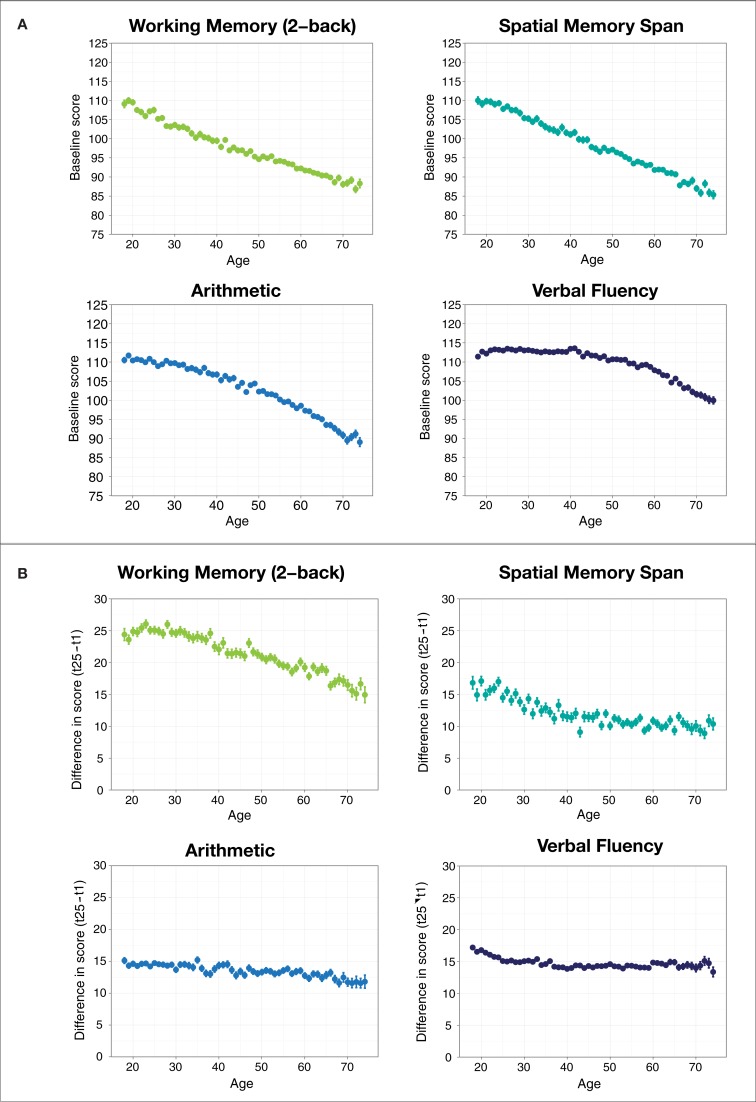
**(A)** Mean game score by age at baseline. **(B)** Difference between 25th and 1st game score by age for each game. Error bars represent standard errors of the mean.

At baseline, we found that performance decreased in all exercises with increasing age, but did so to a greater degree for the exercises thought to rely more on fluid intelligence than those that rely more on crystallized knowledge (Figure 4A). This finding is in line with other research that has found that processing speed and memory span decline earlier than verbal fluency and crystallized intelligence, which are preserved until later in life (Park, 1999; Salthouse, 2009).

As we noted earlier, one particular advantage of the dataset is that it contains information about changes in users' in performance over the course of many training sessions. While users showed improvements in all four tasks with training, the effect was largest for the 2-back working memory task, and smallest for spatial memory span (Figure 4B), perhaps indicating differences in the difficulty and novelty of these tasks. The working memory task also showed the largest negative age-related difference in training improvement, while improvements in verbal fluency remained relatively constant across ages. We also observed that when taken together, training improvements on working memory tasks were less affected by a user's age than performance on the verbal fluency and arithmetic tasks. This provides preliminary evidence that the ability to improve at a task changes with age in the same way that baseline ability changes with age—with an earlier and more rapid effect of age on learning for tasks that rely on fluid intelligence and a more gradual influence of age for tasks that rely more on crystallized knowledge. This finding also runs counter to the theory that individuals who have more initial difficulty with a particular type of task should show greater improvement with training at that task compared to ones that they find easier, based on the idea they have more room for improvement. We found instead that older individuals, who start with lower performance on fluid intelligence tasks, also show slower rates of improvement with training compared to those that rely to a greater degree on crystallized knowledge.

## General discussion

While this initial glimpse at this dataset hints at the potential for very large datasets to provide unique insights to our understanding of human cognition, there are also challenges and potential limitations to the approach taken here. For one, since users are free to train in a variety of more or less controlled ways on the website, our ability to control the training experiences of our samples is reduced when compared to controlled laboratory-based experiments. As with any self-selected online population, the demographics of the Lumosity user base may not perfectly mirror the population, and it isn't currently possible to fully verify users' self-reported health and demographic information.

In the future it will be important complement the “big data” approach taken here with more controlled studies that can further validate these findings. The ability to deliver training and testing online, and the large existing user base offers promise for conducting large-scale controlled experiments that would not be possible in traditional laboratory research. Lumos Labs researchers and external research collaborators are currently designing and running several such studies in different settings, including in schools and in specific patient populations. Making it possible to run controlled experiments on subsets of this user population who have opted in to experimental training will also be crucial to determining the factors underlying peak cognitive performance.

We have only scratched the surface of what the further study of this dataset might uncover, and we would like to invite other researchers interested in questions related to health and cognition to partner with us in exploring our growing dataset in order to make new breakthroughs.

### Conflict of interest statement

Daniel Sternberg, Kacey Ballard, Joseph Hardy and Michael Scanlon are employed by Lumos Labs, and have stock options with the company. Benjamin Katz was also employed by Lumos Labs at the time he contributed to this project. P. Murali Doraiswamy has received research grants and/or advisory fees from NIH and several pharmaceutical companies (for other studies), and he owns stock in Sonexa and Clarimedix (whose products are not discussed here). He has no financial relationship with Lumos Labs.

### Use of Human Subjects Data

The analyses of aggregated de-identified data reported in this manuscript met the criteria for “exempt research” as determined by the Duke University Medical Center Institutional Review Board.
